# Supplementary material for: A Critical Role of the IL-22–IL-22 Binding Protein Axis in Hepatocellular Carcinoma
Source: Cancers (Basel). 2022 Dec 7;14(24):6019. doi: 10.3390/cancers14246019 (PMC9775560; doi:10.3390/cancers14246019)
Supplement: Supplementary file 1 [file cancers-14-06019-s001.zip › cancers-2008339 Supplementary.pdf]

Supplementary Figures

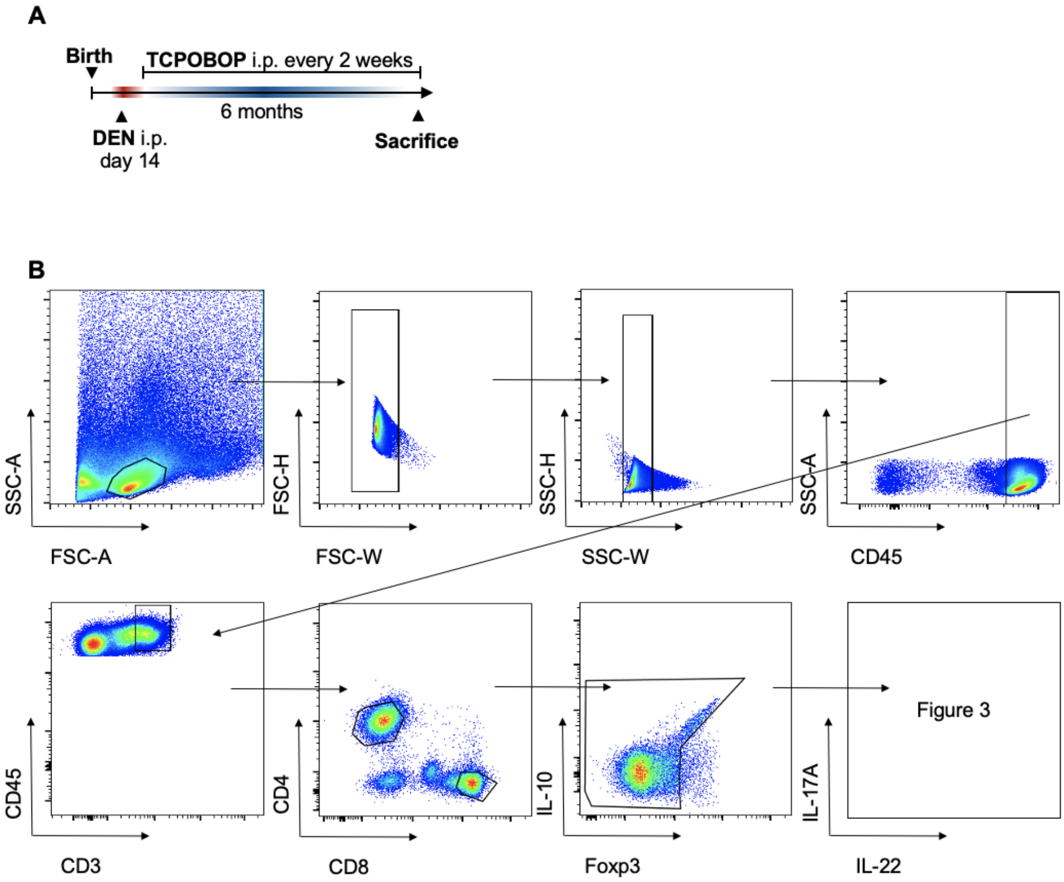

Supplementary Figure S1. A gating strategy for leucocyte analysis with FACS in a chemical HCC model. (**a**) Schematic timeline describing chemical HCC induction with DEN and TCPOBOP in mice; (**b**) Representative dot plots displaying the used gating strategy.

Supplementary Tables

Table S1. Fluorochrome-labelled antibodies used for FACS.

Epitope	Origin	Clone	Fluoro-chrome	Dilution	Manufac-turer
CD45.2	mouse	104	BV786	1:200	Biolegend
CD45.2	mouse	104	Pe-Cy7	1:200	Biolegend
CD3	rat	17A2	BV700	1:100	BioLegend
CD3	rat	17A2	FITC	1:200	BioLegend
CD3	rat	17A2	PE	1:200	BioLegend
CD4	rat	GK1.5	BUV395	1:200	BioLegend
CD4	rat	GK1.5	APC	1:200	BioLegend
CD4	rat	GK1.5	BV605	1:200	BioLegend
CD8	rat	53-6.7	Pe-Cy7	1:200	BioLegend

CD8	rat	53-6.7	APC	1:200	BioLegend
CD11b	rat	M1/70	APC-Cy7	1:200	BioLegend
CD11c	hamster	HL3	FITC	1:200	BD Bioscience
Ly6G	rat	1A8	BV421	1:200	BD Bioscience
Ly6C	rat	AL-21	PE	1:600	BD Bioscience
MHCII	rat	M5/114.15.2	PerCP	1:400	BioLegend

Table S2. Probes used for murine RNA quantification via qPCR.

Gene	Probe name
Cebpd	Mm00843434_s1
Fga	Mm00802584_m1
Fgb	Mm00805336_m1
Hprt1	Mm03024075_m1
Il22	Mm01226722_g1
Il22bp	Mm01192969_m1
Il33	Mm00505403_m1
Steap4	Mm00475405_m1

Table S3. Probes used for human RNA quantification via qPCR.

Gene	Probe name
CEPBD	Hs00270931_s1
FGA	Hs00241027_m1
FGB	Hs00905942_m1
HPRT1	Hs02800695_m1
IL22	Hs01574154_m1
IL33	Hs00369211_m1
STEAP4	Hs01026584_m1
